# The Basel Face Database: A validated set of photographs reflecting systematic differences in Big Two and Big Five personality dimensions

**DOI:** 10.1371/journal.pone.0193190

**Published:** 2018-03-28

**Authors:** Mirella Walker, Sandro Schönborn, Rainer Greifeneder, Thomas Vetter

**Affiliations:** 1 Department of Psychology, University of Basel, Basel, Switzerland; 2 Department for Mathematics and Computer Science, University of Basel, Basel, Switzerland; Bournemouth University, UNITED KINGDOM

## Abstract

Upon a first encounter, individuals spontaneously associate faces with certain personality dimensions. Such first impressions can strongly impact judgments and decisions and may prove highly consequential. Researchers investigating the impact of facial information often rely on (a) real photographs that have been selected to vary on the dimension of interest, (b) morphed photographs, or (c) computer-generated faces (avatars). All three approaches have distinct advantages. Here we present the Basel Face Database, which combines these advantages. In particular, the Basel Face Database consists of real photographs that are subtly, but systematically manipulated to show variations in the perception of the Big Two and the Big Five personality dimensions. To this end, the information specific to each psychological dimension is isolated and modeled in new photographs. Two studies serve as systematic validation of the Basel Face Database. The Basel Face Database opens a new pathway for researchers across psychological disciplines to investigate effects of perceived personality.

## Introduction

First encounters are known to strongly influence how individuals perceive others; the power of “first impressions” is even part of collective wisdom represented in fiction and proverbs (e.g., “You never get a second chance to make a first impression”). One particular prominent source affecting first impressions is the human face. Individuals use facial information to instantly and spontaneously build first impressions about others (e.g., [1, 2, 3]). For instance, they infer childlike traits, such as naiveté to strangers with a babyfaced appearance [4]. Such face-based personality impressions have been shown to influence judgments and behavior in various applied domains, such as mate selection (e.g., [5]), hiring (e.g., [6]), voting (e.g., [7, 8]), jurisdiction (e.g., [9]), and morality (e.g., [10, 11]). Because personality impressions based on faces show a high level of agreement across individuals (e.g., [12, 13]), they may result in a socially shared reality that is highly consequential for those being judged.

Given the impact of facial appearance on personality impressions, it is not surprising that researchers across domains have become interested in using faces as stimuli. Selecting or creating such stimuli to investigate the impact of facial appearance on personality impressions and subsequent judgments and decisions in a given context is a critical part of these endeavors. One approach is to use real photographs as stimuli and to select these in such a way that they differ regarding the cues associated with the personality dimension under investigation. For instance, from a pool of photographs, researchers may select those that were previously rated as especially low versus high on the trait of interest. One caveat with this approach, however, is that there is no control over confounding variables irrelevant for the ascription of that trait, such as the roundishness of the faces, the size of the eyes, or the skin tone, thus putting internal validity at stake. To illustrate, consider a researcher who aims to systematically investigate the impact of facial cues of extraversion on voting behavior. If he or she presents participants in one condition with photographs of political candidates looking introverted and in the other with photographs of candidates looking extroverted, he or she cannot be confident that the differences in voting behavior are in fact due to the differences in facial cues relevant for impressions of the candidates’ extraversion, because the candidates might have also differed on various other facial characteristics that are irrelevant for impressions of extraversion, but are still predictive of voting behavior.

**Fig 1:**
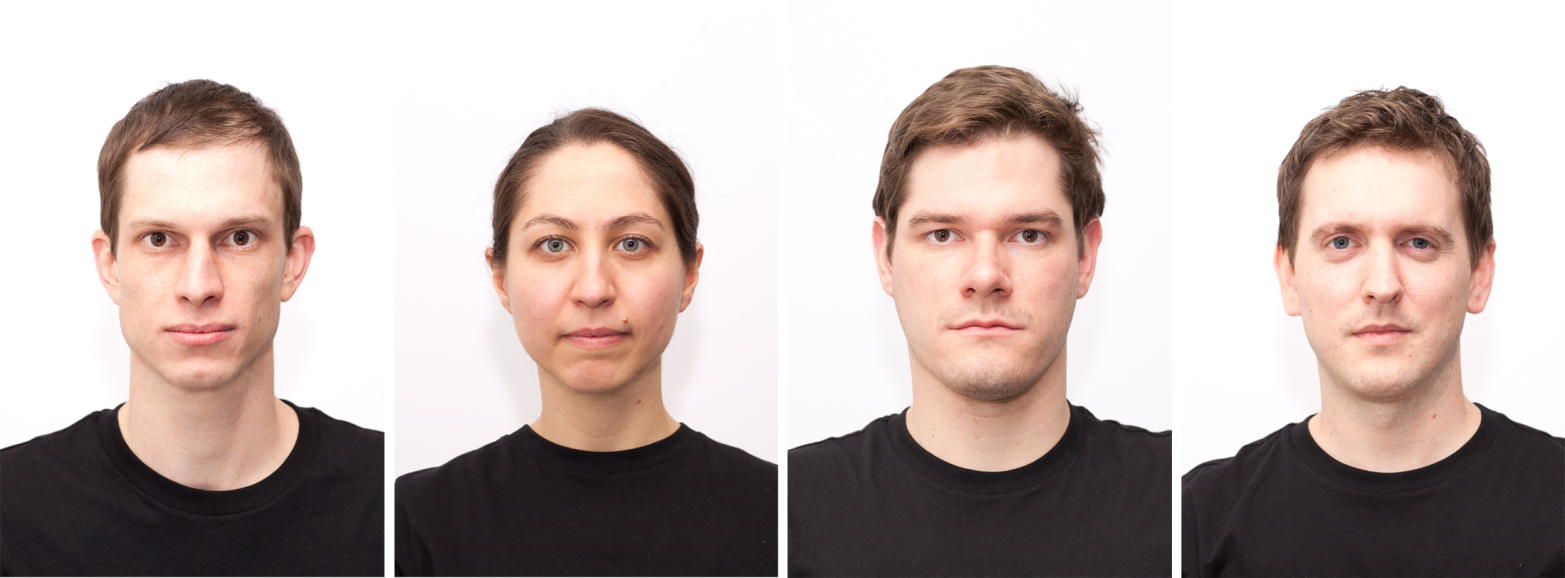
Four exemplar identities of the Basel Face Database.

A second approach was recently introduced by Sutherland and colleagues [14], who use real photographs, but morph these by applying an image-based manipulation technique. This approach deconfounds the information perceived to be systematically associated with the extreme endpoints of a personality dimension from other, irrelevant information (see [15, 16] for methodologically similar procedures focusing on actual rather than on perceived personality dimensions). In order to use this approach to create new realistic stimuli, researchers have successfully met two critical challenges. On the one hand, as a result of morphing, the images have a somewhat blurry look, especially in the forehead region and with regard to extra-facial features, such as hairstyles and clothing. For instance, because hairstyles differ in many respects, and more or less overlap with parts of the face, morphing over hairstyles creates some level of blurriness. Perceivers hence recognize the images as digitally altered (see [14], Fig 1). This limitation proves non-significant if extra-facial features are tightly controlled (e.g., all persons tie their hair back) or if masks are used to remove extra-facial information [17]. On the other hand, the facial information found to be specific for the perception of a certain personality dimension is visualized based on the photographs perceived as extreme on the respective personality dimension. Beyond this visualization, this facial information needs to be systematically isolated, and transferred to novel photographs of faces [18]. Taken together, this second approach allows creating realistic stimuli that systematically vary on personality, to the extent that the above challenges are simultaneously met. In particular, it is critical to reduce blurriness and to systematically isolate and transfer the information found to be specific for the perception of a certain personality dimension to novel photographs of faces.

A third approach could be to present participants with computer-generated facial stimuli that only differ with regard to the facial information relevant for the perception of the personality dimension of interest, while holding all information irrelevant for the perception of that personality dimension constant (e.g., [19]). The advantages of this approach are that the dimensions in question are manipulated in a very controlled way and that the same facial information (e.g., the information relevant for the perception of extraversion) can be applied to an infinite number of different (computer-generated) facial identities. The caveat here is that such stimuli look like avatars and not real face photographs. To the extent that perceived artificialness is an issue, this material may have unwanted consequences and threaten internal and/or ecological validity. Suggesting that perceived artificialness matters, individuals perform the same task differently depending on whether they are presented with computer-generated avatars or real photographs [20]. Note that we do not question the usefulness of avatars per se, but rather suggest that both avatars and photographs may be more or less suitable depending on the question of interest.

All three approaches reviewed above come with distinct advantages and disadvantages. The here provided Basel Face Database (BFD) builds upon a fourth approach, which unites the major advantages, by combining face modeling with image manipulation techniques. This approach is the first to allow modeling of the specific facial information associated with key personality dimensions in novel face photographs of different individuals (see Fig 1 for exemplar photographs). The results of this modeling are realistic looking, non-blurry stimuli.

More specifically, the BFD results from a procedure that systematically, but subtly varies one aspect of a face, while holding all other aspects constant. The technique builds upon previous research [13, 21, 22, 23] using the idea of a face space [24]. The statistical face space [25] is derived from the analysis of real 3D face scans [26]. The dimensions of the space are defined by the information on which these faces maximally vary with regard to both shape and texture. Thus, every individual face can be located in this space and is represented by a linear combination of the resulting dimensions. Collecting personality judgments for these faces allows computation of the dimensions in the face space with maximum variability regarding these personality dimensions. These dimensions are deconfounded from information irrelevant for the ascription of the respective personality dimensions, which also means that they are deconfounded from information specific to one person. Subsequently, any face in this face space can be shifted along any of these dimensions, resulting in faces appearing more or less extreme regarding the respective personality dimensions. Importantly, image manipulation techniques allow application of this method to photographs of real faces without producing visible artifacts. Any face as depicted on a 2D photograph can be actively reconstructed based on the 200 face scans of our face model. It can then be shifted along the personality dimensions and finally–be rendered back into the original photograph.

Here we apply this procedure to systematically model perceptions of the Big Two and Big Five personality dimensions in photographs of 40 different individuals. The Big Two and the Big Five are not the only ways to conceptualize personality, but we focus on these two concepts for the following reasons: The Big Two personality construct with the dimensions communion and agency [27, 28] seems to be particularly important for two reasons: First, agency and communion are semantically similar to the two fundamental dimensions of face evaluation, dominance and trustworthiness, that have been previously found to account for more than 80% of variance in a very diverse set of personality ascriptions [21]. Moreover, these two dimensions are related to the two dimensions competence and warmth to describe social groups in the Stereotype Content Model [29, 30]. Thus, the two dimensions communion (or trustworthiness/warmth) and agency (or dominance/competence) seem to be especially important when characterizing individuals or social groups.

The Big Five personality concept with the dimensions openness to experience, conscientiousness, extraversion, agreeableness, and neuroticism [31, 32] is employed because the Big Five concept is ubiquitous in a variety of applied settings from consumer behavior [33] to mate selection [34] or parenting [35]. Moreover, it has been previously shown that individuals make such fine-grained personality judgments from faces. More importantly, previous work has shown that these five dimensions dissociate in face space, meaning that although these dimensions are correlated to different degrees, every dimension triggers the corresponding personality associations most strongly [23].

In sum, the BFD consists of 40 real face photographs that are systematically manipulated regarding the perception of the Big Two and the Big Five personality dimensions, thus allowing for high ecological and internal validity. Researchers across disciplines interested in the impact of personality cues in faces on judgments and decisions may fruitfully rely on this database. In what follows, we first describe how the database was developed. Then, we report validation studies separately for the Big Two and the Big Five personality dimensions.

This research project was approved by the Institutional Review Board of the Department of Psychology at the University of Basel (IRB approval No. 034-15-3). The individuals in this manuscript have given written informed consent (as outlined in PLOS consent form) to publish their portraits.

## Database development

### Target sample

The target sample consists of 40 undergraduate students (18 male, 22 female; *M*_age_ = 23.23, *SD*_age =_ 3.23) from the University of Basel. First, the students signed a consent form in which they indicated that they allow us and other researchers to use their portrait and variations of it for research purposes and indicated whether they also agree with the publication of their portrait and variations of it in scientific publications, which 30 participants did. Then participants were asked to put on a black t-shirt and to pull their hair back. Participants were instructed to sit straight on a chair in front of a white wall with a neutral, relaxed facial expression and to directly gaze at the camera. Their picture was taken with a Canon EOS 5D camera with a 85mm lens and a Nissin Di622 flash unit and saved in the RAW format. The distance from the chair-back to the camera was 1.80 meters.

### Photo-editing

**Fig 2:**
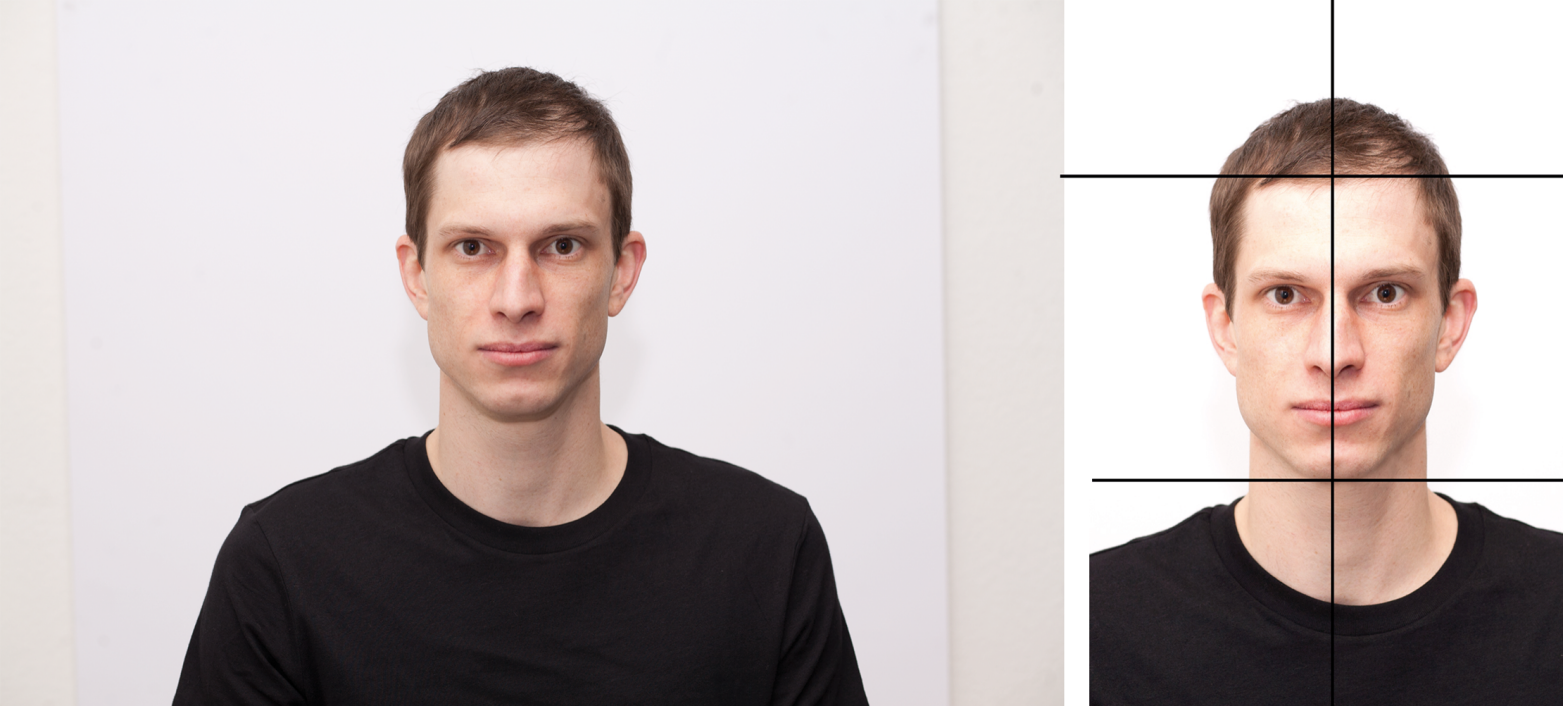
The RAW image (left) was edited so that the background color was standardized, the axis between the eyes was centered, and the face size was fixed by aligning the chin and hairline (right).

Photographs were edited with Adobe Photoshop CS5. If necessary, lightness was adjusted so that the background colors of all photographs looked similar. Then, each portrait was horizontally centered and the sizes of the faces were fixed by aligning both chin and hairline (see Fig 2). In one photograph, the t-shirt had to be digitally edited, because the neckline was considerably lower than in the other photographs. Photographs were exported as JPG images (see Fig 1 for exemplar photographs).

### Face modeling

Systematically manipulating the perceived personality in novel 2D photographs of faces involves three steps: First, we *locate* the photographed faces in the Basel Face Model. Second, we *shift* the faces along the dimensions of the Basel Face Model, which correspond to the perception of specific personality dimensions. Third, we *render* the resulting faces back into the original photograph to obtain natural-looking results.

**Fig 3:**
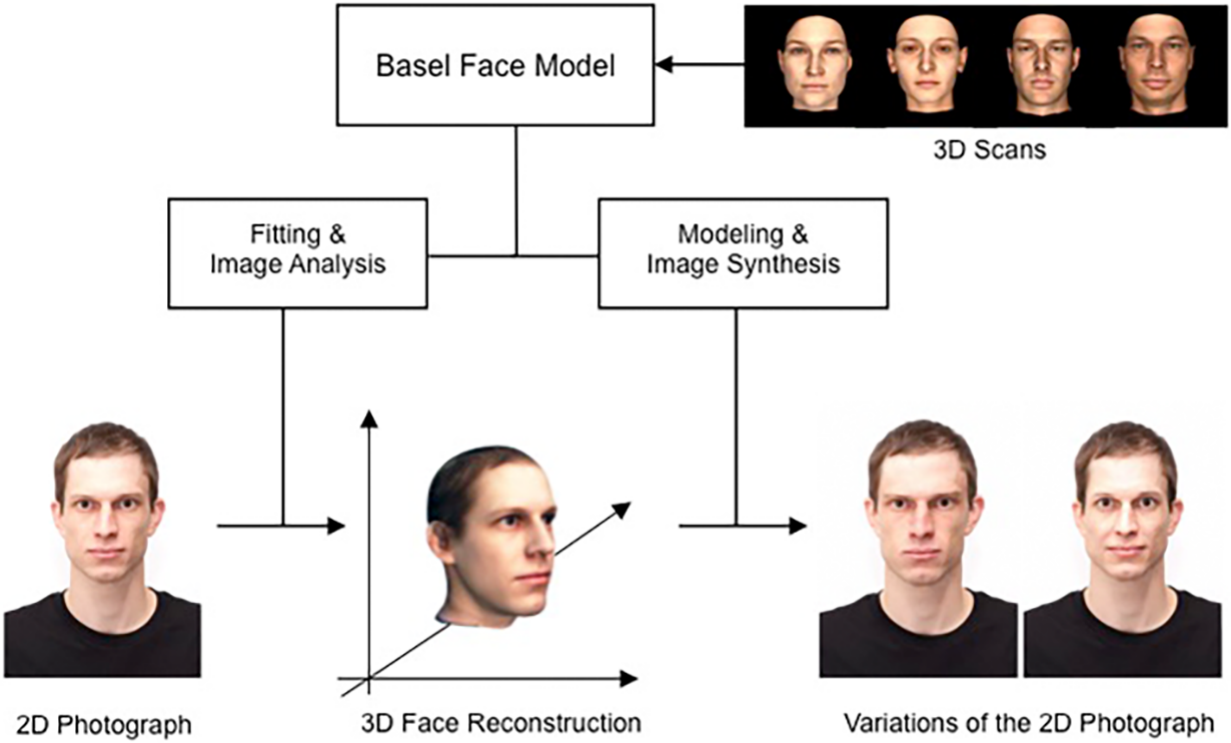
Visualization of the process to generate variations of an exemplar face on a specific personality dimension (here: Agreeableness). First, the face on the standardized and aligned photograph is reconstructed by fitting the Basel Face Model [26] to the face on the photograph. Second, the resulting 3D reconstruction of the face is manipulated on a specific personality dimension. Finally, the altered faces are rendered back into the 2D photograph, resulting in natural-looking images that vary regarding their value on the respective personality dimension.

The Basel Face Model is based on 200 3D scans of real faces. The dimensions of this model describe the properties on which these faces vary. The closeness/distance between different faces in this model reflects the similarity/difference between them. The closer two faces in the model, the more similar these faces are. Locating the photographed faces on the dimensions of the Basel Face Model (i.e., Step 1) is achieved by actively reconstructing these faces using the 200 3D scans the model is built upon (see [36] for detailed information about these 200 faces and the model built from them). In Step 2, the reconstructed faces are shifted along the dimensions that reflect perceived Big Two and Big Five. This shifting requires that the relations between the dimensions of the model and the perception of the different personality dimensions of interest have been defined. We defined these relations empirically by collecting Big Two and Big Five judgments for the majority of the 3D scans the model is based upon, and then linearly regressing the dimensions of our face model to the averaged personality judgments. As a result of this procedure, the regression coefficients describe the dimensions in the face model that explain most variance in the Big Two and the Big Five personality judgments und thus correspond to perceptions of these personality dimensions (see [23]). Note that in face space, the Big Two dimensions are almost perfectly independent from each other (*r* = -.02), whereas the Big Five dimensions are not, because the underlying personality judgments are correlated to different degrees (.04 < *|r|* < .77). We decided not to deconfound those dimensions in the face space, because we aimed for maximally natural-looking models. If judgments of conscientiousness and openness, for example, go hand in hand to a certain degree, meaning that some facial information used to make conscientiousness judgments is also used to make openness judgments, we want to have this facial information in the conscientiousness and in the openness model. In short, our aim is to systematically isolate all the facial information that shapes the perception of a specific personality dimension (even if this information also shapes the perception of another dimension) from all the facial information irrelevant for the perception of that specific personality dimension (i.e., information specific to one person). Importantly, it has previously been shown that although the different dimensions are not orthogonal to each other, individuals are able to distinguish them from each other [23]. For every face we created 14 new versions, five with a reduced and five with an enhanced value on each single Big Five dimension and two with a reduced and two with an enhanced value on both Big Two dimensions. Note that when using the term value we refer to the manipulated perceived (i.e., ostensible rather than actual) personality. Rendering these altered faces back into the edited photographs (i.e., Step 3) resulted in natural-looking stimuli (see Fig 3 for a schematic overview of that procedure).

## Database validation study 1 –Big Two

### Method

#### Participants

We recruited 193 participants (~32 participants per face; *M*_age_ = 39.78, *SD* = 11.79) from Amazon Mechanical Turk (96 male, 97 female). Sample size was determined using Pangea [37].

#### Design

The design was a 2 (Personality dimension: Agency vs. Communion; between-participants) x 3 (Face value: Reduced, Original, and Enhanced; within-participants) design with the dependent variables ascribed personality.

#### Material

To measure the Big Two personality inferences we translated the five items with the highest factor loadings per scale from the German Personal Attributes Questionnaire (GEPAQ; [38]) that we initially used to develop the Big Two vectors [23] into English. They were reframed in order to assess personality traits of others instead of oneself. For example, to evaluate agency, participants indicated to what extent the person depicted “is active” on a scale from 1 = “does not apply at all” to 5 = “fully applies”.

**Fig 4:**
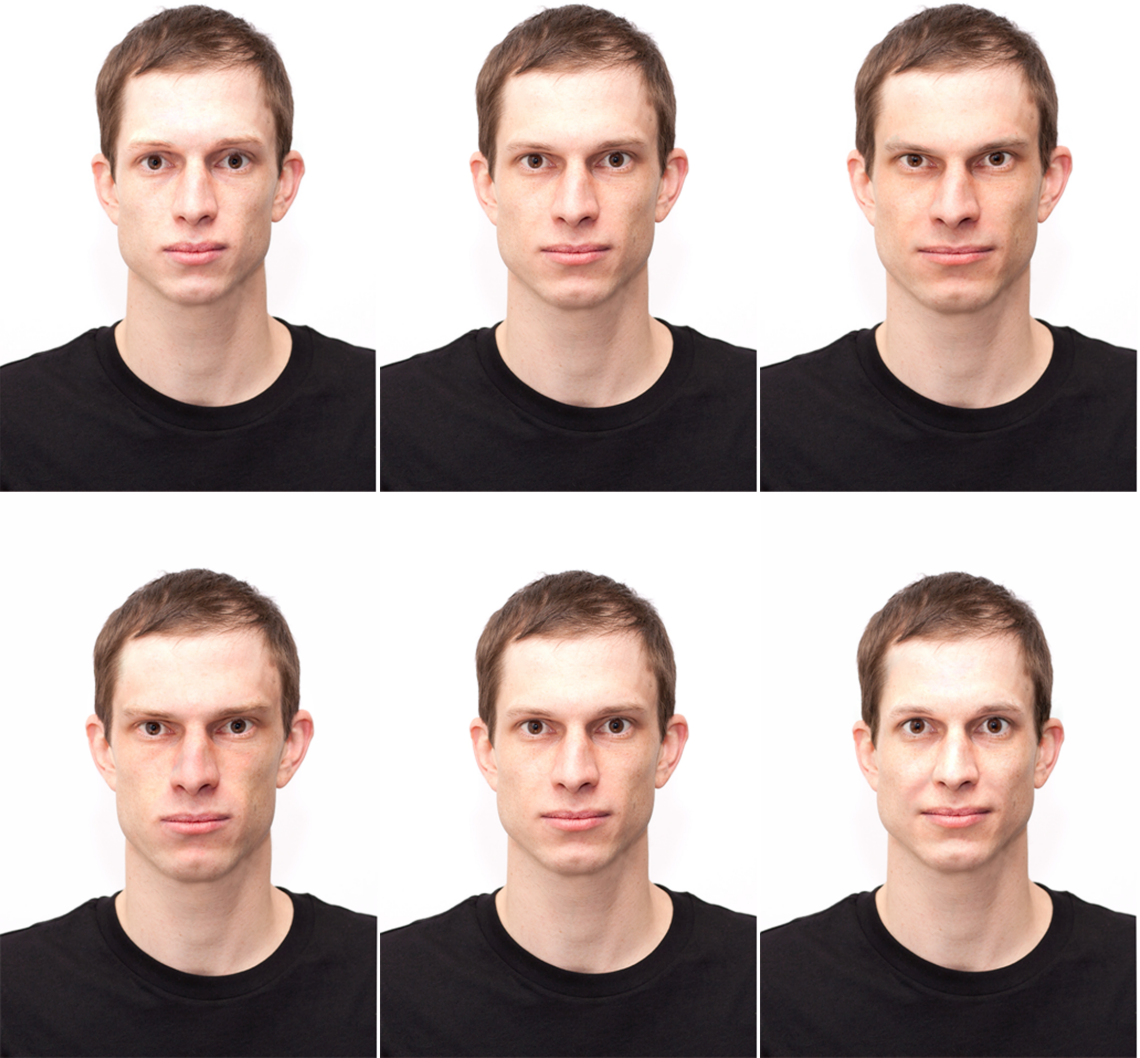
An original face (middle) and versions with a reduced (left) and an enhanced value (right) on agency (top) and communion (bottom).

The stimuli were the 40 original faces and 40 versions with an enhanced value on agency, 40 versions with an enhanced value on communion, 40 versions with a reduced value on agency, and 40 versions with a reduced value on communion, resulting in a total of 200 faces (see Fig 4 for one exemplar face). Because on the one hand, every participant should be presented with one stimulus person only once (either with the original version, the version with a reduced or the version with an enhanced value on one of the two dimensions) and on the other hand, every face should be evaluated in every version, we divided the 40 stimulus persons into three sets of 13, 13, and 14 stimuli. Participants, for example, saw all faces in set 1 in the original version, all faces in set 2 with an enhanced value and all faces in set 3 with a reduced value on the personality dimension in question. Presentation of sets to participants was counterbalanced by a Latin square.

#### Procedure

Participants were welcomed and told that the study was investigating personality inferences based on limited information, namely portraits of strangers. Before starting with the study, participants were asked to provide informed consent. Participants were randomly assigned to one of two personality conditions (agency vs. communion). Within both conditions, each participant saw one set of faces in the original version, one set with an enhanced value, and one set with a reduced value on the respective personality dimension. All participants worked on all three sets, that is, all 40 faces. Presentation of faces was random. Participants were asked to judge the 40 faces on 4 items capturing either agency or communion. Participants were debriefed and explicitly told that some portraits had been digitally altered to change the perception of personality, followed by an item measuring the originality/manipulatedness of the 40 portraits they had seen before (S1 Supporting Information). This data was collected for a different project that investigates the relation between the averageness/uniqueness of perceived personality characteristics and the perceived originality/manipulatedness of the respective portrait. Finally, participants provided demographical information, were thanked, and given a code in order to get paid by Amazon Mechanical Turk.

### Results

Agency and communion items were combined to reliable agency and communion scales (Cronbach’s alphas > .900). We analyzed our data using linear mixed models analyses including random effects for participants and faces. The advantage of this method is that it allows the generalization of results across both participants and faces [39].

To test whether our method to manipulate the perception of agency and communion was successfully applied to the 40 faces, we fitted different linear mixed models to our data. We aimed for maximal linear mixed models because such models generalize best across participants and stimuli [40]. Therefore, we included a random intercept (i.e., the model allows the intercept to vary individually) and a random slope for the main effect of face value based on participants (i.e., the model allows the face value to individually affect different participants’ judgments) as well as a random intercept and random slopes for both main effects (i.e., Face value and Personality dimension) based on faces (i.e., the model allows the intercept to vary individually and face value and personality dimension to individually affect different faces) in all models presented. Data were analyzed using the lme4 package [41] in R [42]. The reason for only including random slopes for face value for participants is that only face value was manipulated within-participants and the reason for not including the interaction term for faces is that the respective models would not converge due to the number of observations.

First we built a model only specifying the random effects as described above (AIC = 17262). Second, we built a face value model by adding the fixed factor face value to the random model (AIC = 17129). Comparing these two models revealed a better model fit for the face value model, c^2^(2) = 136.79, *p* < .001, indicating that manipulating the face value on personality dimensions had a significant impact on personality judgments. Faces with a reduced value on the Big Two dimensions were ascribed lower levels on these personality dimensions (*M =* 2.52, *SE =* 0.06) than the original faces (*M =* 3.27, *SE =* 0.07), which were ascribed lower levels than the faces with an enhanced value (*M =* 3.62, *SE =* 0.06).

Third, we tested whether this main effect of face value was qualified by an interaction effect with the factor personality dimension. Therefore, we added the main effect personality dimension and the interaction term to the model (AIC = 17108). The comparison of the face value model with the interaction model revealed a better fit for the interaction model model, c^2^(3) = 26.66, *p* < .001, indicating that the manipulation worked better for communion than for agency.

**Fig 5:**
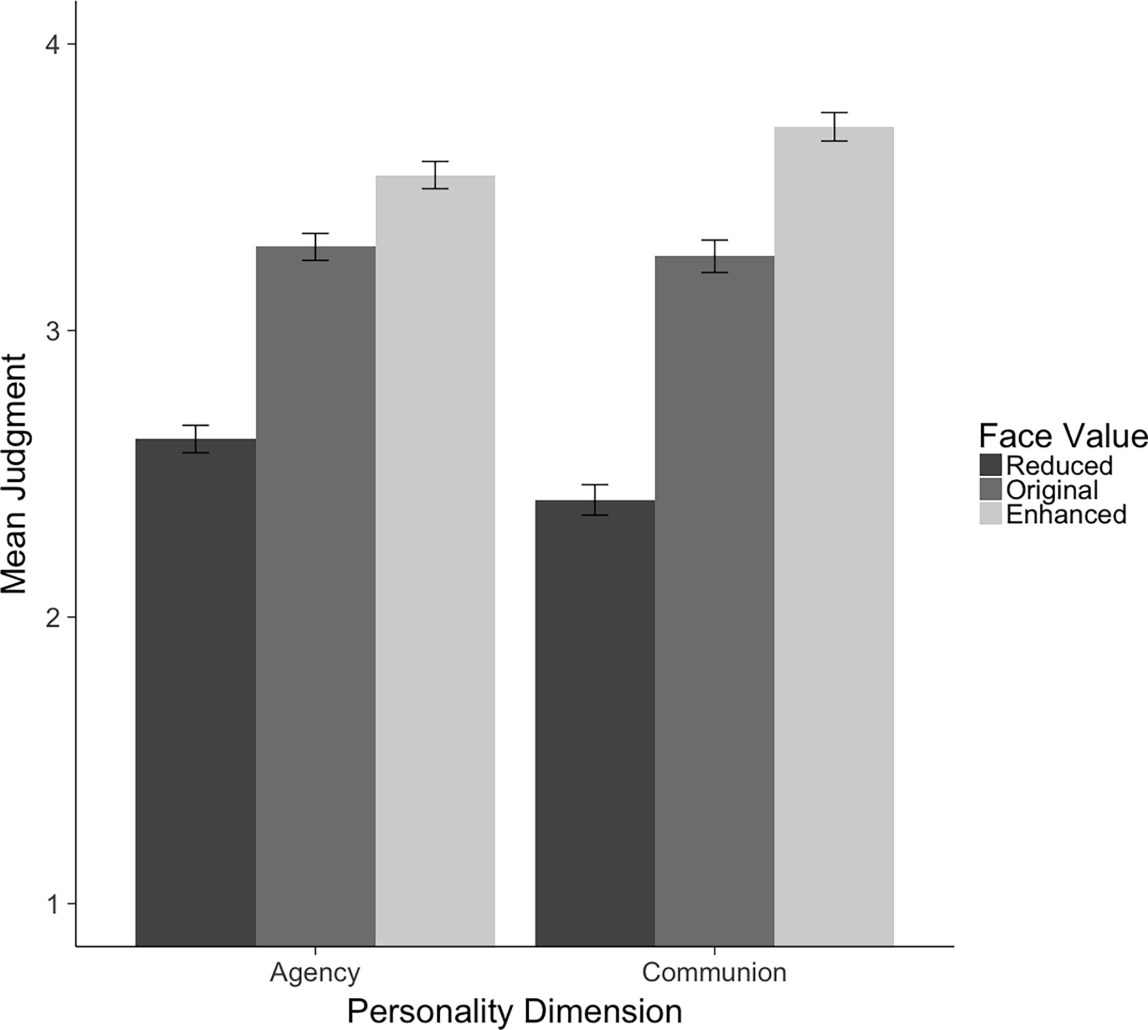
Means (and 95% CIs) for personality judgments collapsed over all 40 database faces with reduced, original, and enhanced values on agency and communion.

Because communion was more strongly affected by the face manipulation than agency, we report communion and agency separately in what follows. Both face value models (AIC_Ag_ = 9069.5, AIC_Com_ = 8034.5) show a significantly better model fit than the respective random models (AIC_Ag_ = 9172.5, AIC_Com_ = 8161.8; c^2^_Ag_(2) = 107.01, *p* < .001, c^2^_Com_(2) = 131.29, *p* < .001. More specifically, we found significant linear trends for both personality dimensions, *t*_*Ag*_ (94) = 13.46, *p* < .001, *t*_*Com*_ (91.02) = 20.37, *p* < .001, indicating that reducing the value on agency or communion in a face leads to lower judgments on the respective dimension, while enhancing the value of agency or communion leads to higher judgments on the respective dimension compared to the original version of the face. Table 1 shows means and standard deviations for reduced, original, and enhanced values on agency and communion, separately for every single face as well as collapsed over all 40 faces. Fig 5 visualizes these effects collapsed over the 40 faces.

**Table 1:** Means and standard deviations of the Big Two judgments for the 40 facial identities with reduced, original, and enhanced values on both Big Two personality dimensions. Please note that the neutral versions (-/+) in the Agency and the Communion columns refer to the exact same stimuli.

ID	Agency			Communion		
	- M(SD)	-/+ M(SD)	+ M(SD)	- M(SD)	-/+ M(SD)	+ M(SD)
1	2.38 (0.95)	3.15 (0.92)	3.74 (0.62)	2.35 (0.96)	3.05 (0.72)	3.68 (0.81)
2	3.03 (0.91)	3.59 (0.83)	4.01 (0.70)	3.15 (1.00)	3.81 (0.84)	4.29 (0.79)
3	2.29 (0.80)	3.10 (0.75)	3.37 (0.83)	2.24 (0.71)	2.96 (0.93)	3.47 (0.79)
4	2.43 (0.88)	3.30 (0.81)	3.88 (0.77)	2.19 (0.85)	2.84 (0.99)	3.58 (0.75)
5	2.75 (0.94)	3.13 (1.08)	3.78 (0.88)	2.13 (0.94)	2.54 (0.96)	3.10 (0.96)
6	3.20 (0.83)	3.80 (0.70)	3.78 (0.77)	3.26 (0.86)	4.20 (0.67)	4.48 (0.53)
7	3.10 (0.90)	3.71 (0.65)	3.70 (0.86)	2.49 (0.95)	3.59 (0.91)	4.23 (0.70)
8	2.45 (0.77)	2.88 (0.84)	3.07 (0.79)	2.73 (0.98)	3.27 (0.95)	3.83 (0.92)
9	2.45 (0.75)	3.46 (0.73)	3.33 (1.06)	1.73 (0.54)	2.58 (0.93)	3.79 (0.86)
10	2.04 (0.91)	2.88 (0.79)	3.47 (0.97)	2.60 (0.97)	3.17 (0.86)	3.57 (0.75)
11	2.64 (0.68)	2.96 (0.76)	3.25 (0.96)	2.35 (1.11)	3.00 (1.11)	3.60 (0.89)
12	2.30 (0.74)	3.30 (0.73)	3.13 (0.92)	2.54 (0.73)	3.78 (0.82)	3.95 (0.80)
13	3.01 (0.75)	3.75 (0.67)	4.02 (0.76)	2.66 (0.97)	3.98 (0.78)	4.31 (0.56)
14	2.51 (0.95)	3.20 (0.88)	3.62 (0.83)	2.54 (0.79)	3.71 (0.85)	4.01 (0.80)
15	2.50 (0.83)	3.42 (0.86)	3.50 (0.94)	2.53 (0.85)	3.38 (1.04)	3.85 (0.73)
16	2.70 (0.82)	3.36 (0.76)	4.10 (0.64)	2.24 (0.91)	3.10 (0.90)	3.60 (0.62)
17	3.03 (0.84)	3.36 (1.03)	3.69 (0.85)	2.86 (1.06)	4.02 (0.84)	4.19 (0.77)
18	2.67 (0.85)	3.48 (0.70)	3.43 (0.84)	2.74 (0.98)	3.67 (0.88)	4.10 (0.72)
19	2.70 (0.88)	3.66 (0.78)	3.61 (0.80)	2.38 (0.97)	3.67 (0.79)	4.04 (0.73)
20	2.66 (0.93)	3.33 (1.12)	3.62 (0.77)	2.91 (0.93)	3.66 (0.97)	3.82 (0.65)
21	2.06 (0.69)	2.66 (0.72)	2.39 (0.61)	1.92 (0.66)	2.45 (0.96)	2.89 (1.09)
22	2.60 (0.81)	3.26 (0.84)	3.26 (1.01)	2.90 (1.08)	3.56 (0.97)	4.00 (0.68)
23	2.32 (0.82)	2.23 (0.86)	2.61 (0.75)	2.32 (0.97)	2.70 (0.94)	3.39 (0.97)
24	2.64 (0.78)	3.73 (0.82)	3.78 (0.85)	2.57 (0.79)	3.82 (0.85)	3.90 (0.86)
25	1.80 (0.82)	2.54 (0.68)	3.20 (0.86)	2.18 (0.96)	3.05 (0.94)	3.44 (0.69)
26	2.41 (0.89)	3.07 (1.05)	3.63 (0.73)	2.78 (0.78)	3.74 (0.81)	4.01 (0.86)
27	3.06 (0.76)	3.56 (0.94)	3.28 (0.97)	2.12 (0.80)	3.08 (0.84)	3.50 (0.98)
28	3.02 (0.97)	3.68 (0.72)	3.99 (0.61)	2.08 (0.96)	2.75 (0.93)	3.80 (0.70)
29	2.90 (1.07)	3.52 (0.83)	3.83 (0.76)	2.31 (0.93)	3.49 (0.96)	4.08 (0.68)
30	2.31 (0.80)	3.11 (0.88)	2.85 (1.02)	2.22 (0.88)	3.14 (1.00)	3.33 (0.99)
31	2.30 (0.94)	3.07 (0.97)	3.81 (0.78)	1.85 (0.86)	2.59 (0.92)	3.25 (0.95)
32	3.28 (0.84)	3.48 (1.05)	3.94 (0.70)	2.37 (0.95)	2.94 (0.88)	3.37 (0.93)
33	2.34 (0.84)	2.90 (0.88)	3.35 (0.91)	2.20 (0.83)	2.79 (0.91)	3.31 (0.90)
34	2.56 (0.96)	3.29 (0.79)	3.46 (0.73)	2.34 (0.91)	2.78 (0.79)	3.49 (0.84)
35	3.09 (0.88)	3.37 (0.94)	3.67 (0.88)	2.58 (0.85)	3.69 (0.91)	3.76 (0.92)
36	2.37 (0.79)	3.20 (0.85)	2.80 (0.88)	2.18 (0.65)	2.94 (1.03)	3.44 (0.88)
37	2.95 (0.84)	3.72 (0.75)	3.90 (0.79)	2.69 (1.01)	3.95 (0.61)	4.12 (0.70)
38	2.67 (0.88)	3.20 (0.85)	3.92 (0.67)	2.48 (0.97)	3.40 (0.79)	3.88 (0.79)
39	2.47 (0.75)	3.47 (0.75)	3.31 (0.97)	2.54 (0.95)	3.38 (0.84)	3.71 (0.79)
40	2.27 (0.90)	3.04 (0.74)	3.52 (0.81)	1.89 (0.86)	2.29 (0.87)	3.07 (0.98)
all	2.61 (1.10)	3.28 (1.09)	3.53 (1.10)	2.42 (1.16)	3.27 (1.23)	3.73 (1.08)

### Discussion

The aim of the present paper is to provide researchers with variations of faces derived from the same 40 face photographs differing in how the respective persons are perceived with regard to their personality. In this first validation study we show that the 40 faces were indeed successfully manipulated regarding the Big Two dimensions agency and communion. These effects were very similar across different faces. Nevertheless, we provide descriptive data for the 40 individual faces separately, so that the most promising identities for a given purpose can be systematically selected. We recommend sampling sets of faces rather than single faces, because this allows for more powerful statistical analyses (see[39]).

## Database validation study 2 –Big Five

### Method

#### Participants

We recruited 481 participants (~32 participants per condition; *M*_age_ = 37.95, *SD* = 12.55) from Amazon Mechanical Turk (242 male, 238 female, 1 undisclosed). Sample size was determined using Pangea [37].

#### Design

The design was a 5 (Personality Dimension: Neuroticism, Extraversion, Openness to Experience, Agreeableness, and Conscientiousness; between-participants) x 3 (Face value: Reduced, Original, and Enhanced; within-participants) design with the dependent variables ascribed personality.

#### Material

To measure the Big Five personality judgments we translated the German 21 item questionnaire that we used to develop the Big Five vectors [43] into English. The items were reframed in order to assess personality traits of others instead of oneself. For example, participants had to indicate to what extent the person depicted “is sociable” on a scale from 1 = “does not apply at all” to 5 = “fully applies”.

**Fig 6:**
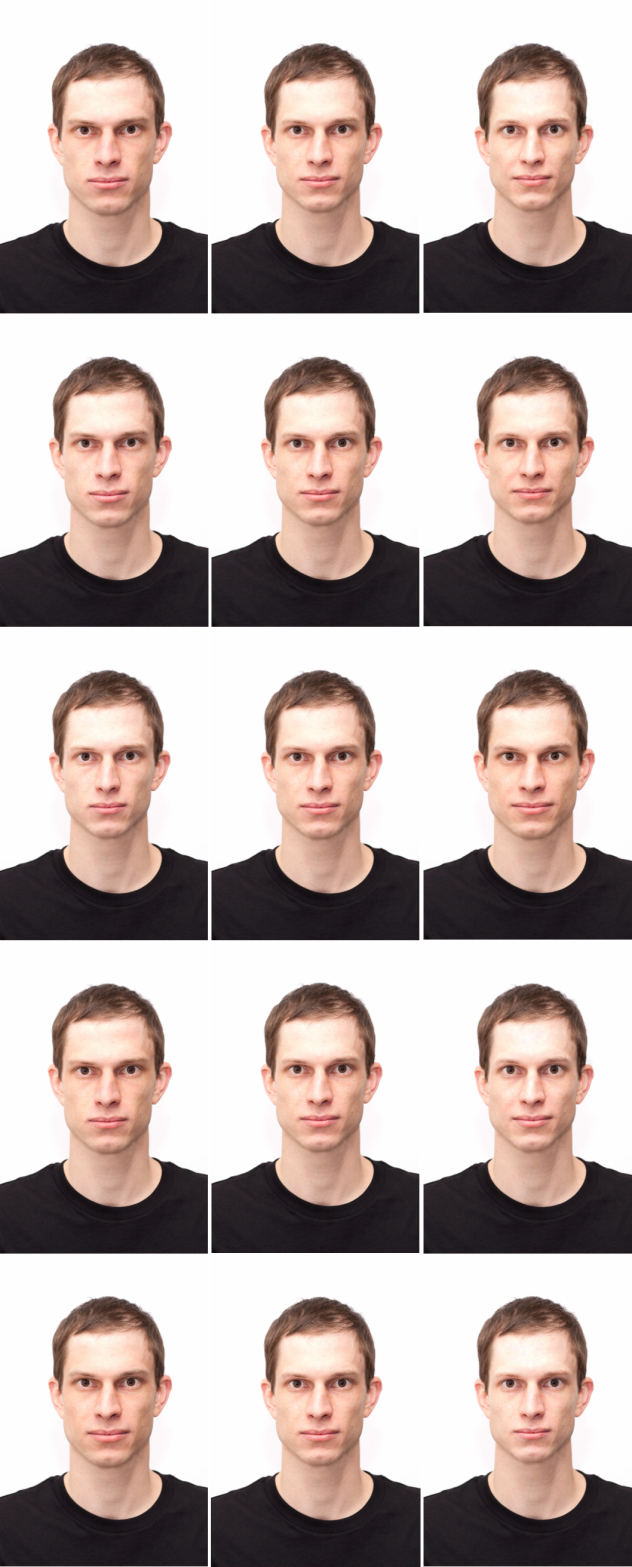
An original face (middle) and versions with reduced (left) and enhanced values (right) on both Big Five personality dimensions (from top to bottom: openness, conscientiousness, extraversion, agreeableness, neuroticism).

The stimuli were the 40 original faces, 200 versions with enhanced values (40 on each of the Big Five dimensions openness to experience, conscientiousness, extraversion, agreeableness, and neuroticism), and 200 versions with reduced values (40 on each of the Big Five dimension). The full set thus consisted of 440 facial stimuli (see Fig 6 for one exemplar face). Faces were divided into sets, which were counterbalanced between participants by a Latin square as in Study 1 so that we could collect ratings for every version of every face without presenting the same participant with the same stimulus person more than once.

#### Procedure

The procedure was the same as in Study 1 with the only exception that participants were randomly assigned to one of five instead of two conditions (i.e., openness, conscientiousness, extraversion, agreeableness, and neuroticism).

### Results

The items used to measure the perception of the different personality dimensions from faces were combined into reliable scales (Cronbach’s alphas > .766). To test whether our method to model the perception of the Big Five personality dimensions was successfully applied to the 40 faces, we analyzed our 3 (Face value: Reduced, Original, and Enhanced; within-participants) x 5 (Personality Dimension: Openness to Experience, Conscientiousness, Extraversion, Agreeableness, Neuroticism; between participants) data with the dependent variable personality judgment using the lme4 function [41] in R [42].

First, we built the maximal linear mixed model [40] suitable for our experimental design by allowing participants and faces to show individual intercepts and slopes for face value and additionally allowing faces to show individual slopes for different personality dimensions (AIC = 46316). Second, we built a face value model by adding the fixed factor face value to the random model (AIC = 46239). Comparing these two models revealed a better model fit for the face value model, c^2^(2) = 81.77, *p* < .001, indicating that manipulating faces regarding their values on personality dimensions had a significant impact on personality judgments. Faces with reduced values on the Big Five dimensions were ascribed lower levels of these personality dimensions (*M =* 2.83, *SE =* 0.03) than the original faces (*M =* 3.06, *SE =* 0.03), which were ascribed lower levels than the faces with enhanced values (*M =* 3.27, *SE =* 0.03).

Third, we tested whether this main effect of face value was qualified by an interaction effect with the factor personality dimension. Therefore, we added the main effect personality dimension and the interaction term to the face value model (AIC = 46152). The comparison of the face value model with the interaction model revealed a better fit for the interaction model, c^2^(12) = 110.45, *p* < .001, indicating that the manipulation worked better for some than for other personality dimensions.

**Fig 7:**
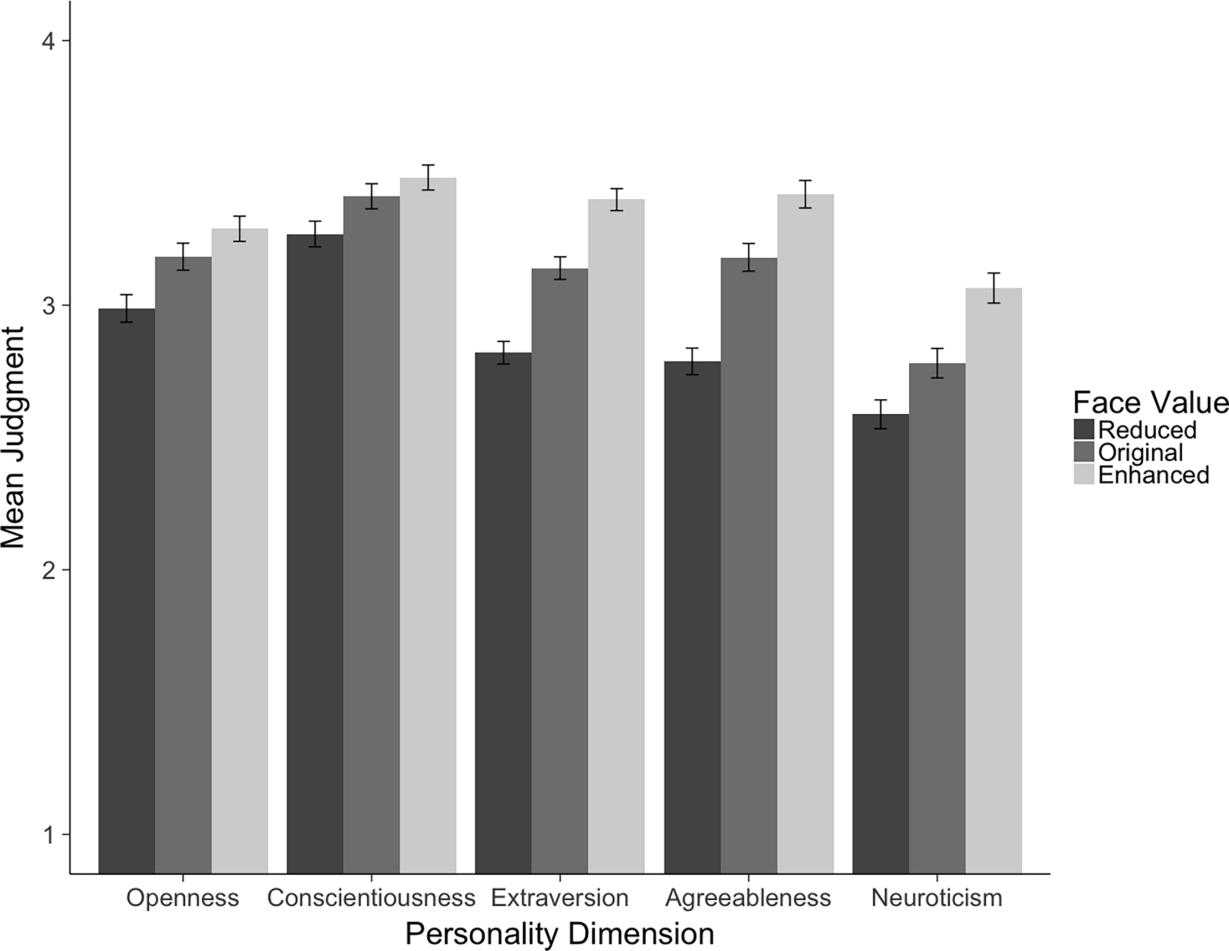
Means (and 95% CIs) for personality judgments for all 40 database faces with reduced, original, and enhanced values on openness, conscientiousness, extraversion, agreeableness, and neuroticism.

Because the different Big Five judgments were differently affected by the face values on the respective traits, we focus on the different personality dimensions separately in what follows. Results show that the face value models (AIC_O_ = 9183.5, AIC_C_ = 9422.4, AIC_E_ = 9362.8, AIC_A_ = 9479.0, AIC_N_ = 8646.9) for all five personality dimensions show a significantly better model fit than the respective random models (AIC_O_ = 9210.6, AIC_C_ = 9440.4, AIC_E_ = 9433.9, AIC_A_ = 9548.6, AIC_N_ = 8690.3; c^2^_O_(2) = 31.07, *p* < .001, c^2^_C_(2) = 21.94, *p* < .001, c^2^_E_(2) = 75.07, *p* < .001, c^2^_A_(2) = 73.57, *p* < .001, c^2^_N_(2) = 47.40, *p* < .001). More specifically, we found significant linear trends for all Big Five dimensions, *t*_*O*_ (51.49) = 6.47, *p* < .001, *t*_*C*_ (58.99) = 4.87, *p* < .001, *t*_*E*_ (74.21) = 11.80, *p* < .001, *t*_*A*_ (70.10) = 11.16, *p* < .001, and *t*_*N*_ (52.28) = 8.21, *p* < .001, indicating that reducing the value on any personality dimension in a face leads to lower judgments on the respective dimension, while enhancing the value on any personality dimension leads to higher judgments on the respective dimension compared to the original version of the face. Comparing the linear trend effects for the five different dimensions reveals the strongest effects for the dimensions extraversion and agreeableness, and the weakest (but still significant) effects for the dimensions openness to experience and conscientiousness. Table 2 shows means and standard deviations for reduced, original, and enhanced values on all Big Five dimensions separately for every single face as well as collapsed over all 40 faces. Fig 7 visualizes these effects collapsed over all 40 faces.

**Table 2:** Means and standard deviations of the Big Five judgments for the 40 facial identities with reduced, original, and enhanced values on all Big Five personality dimensions. Please note that the neutral versions (-/+) of all five personality dimensions refer to the exact same stimuli.

ID	Openness			Conscientiousness			Extraversion			Agreeableness			Neuroticism		
	- M(SD)	-/+ M(SD)	+ M(SD)	- M(SD)	-/+ M(SD)	+ M(SD)	- M(SD)	-/+ M(SD)	+ M(SD)	- M(SD)	-/+ M(SD)	+ M(SD)	- M(SD)	-/+ M(SD)	+ M(SD)
1	2.63 (0.80)	3.15 (0.83)	3.33 (1.02)	2.77 (0.93)	3.02 (0.84)	2.79 (1.01)	2.82 (0.92)	3.28 (0.70)	3.55 (0.81)	2.67 (1.00)	3.12 (0.99)	3.45 (0.86)	2.68 (0.72)	2.54 (0.88)	3.00 (0.91)
2	3.17 (0.77)	3.50 (0.89)	3.71 (0.72)	2.97 (0.91)	3.42 (0.80)	3.32 (0.88)	3.34 (0.71)	3.72 (0.68)	3.85 (0.46)	2.75 (0.78)	3.25 (0.95)	3.94 (0.69)	2.48 (0.77)	2.59 (0.80)	2.53 (1.08)
3	2.68 (0.88)	2.91 (0.96)	3.11 (0.77)	2.44 (0.86)	2.70 (0.87)	3.01 (0.64)	2.90 (0.85)	3.16 (0.73)	3.12 (0.76)	2.74 (0.77)	2.63 (0.91)	3.08 (0.93)	2.68 (1.05)	3.15 (0.84)	3.58 (0.86)
4	2.86 (1.08)	3.17 (0.58)	3.50 (0.81)	2.83 (0.83)	2.42 (0.72)	2.51 (0.93)	3.21 (0.83)	3.33 (0.74)	3.85 (0.82)	2.60 (0.88)	3.00 (0.82)	3.34 (1.08)	3.02 (0.88)	2.88 (0.84)	3.38 (1.08)
5	2.57 (0.98)	3.38 (0.77)	3.16 (0.74)	3.05 (0.83)	3.41 (0.68)	3.44 (0.76)	2.62 (0.77)	3.06 (0.80)	3.11 (0.80)	1.97 (0.72)	2.78 (1.02)	3.38 (0.96)	2.87 (0.81)	3.11 (0.72)	3.49 (0.93)
6	3.36 (0.88)	3.71 (0.61)	4.02 (0.63)	3.45 (0.70)	3.31 (0.89)	3.40 (0.58)	3.20 (0.77)	3.44 (0.74)	3.56 (0.76)	3.48 (0.90)	3.69 (0.89)	4.19 (0.68)	2.71 (1.17)	2.58 (0.86)	2.70 (0.83)
7	3.36 (0.91)	3.78 (0.98)	3.49 (0.74)	3.27 (0.77)	3.53 (0.70)	3.58 (0.87)	3.02 (0.74)	3.07 (0.67)	3.50 (0.76)	2.91 (0.87)	3.27 (0.72)	3.48 (1.09)	2.97 (0.68)	3.26 (0.99)	2.89 (0.85)
8	2.66 (0.87)	3.28 (0.85)	3.35 (0.83)	3.26 (0.84)	3.45 (0.81)	3.39 (0.81)	2.63 (0.76)	2.92 (0.77)	3.14 (0.67)	3.01 (0.84)	3.38 (0.70)	3.55 (0.81)	3.35 (0.87)	3.35 (0.76)	3.69 (0.73)
9	2.33 (0.79)	2.93 (0.91)	3.16 (0.59)	2.71 (1.03)	2.68 (0.90)	3.06 (0.95)	2.42 (0.56)	3.21 (0.88)	3.57 (0.65)	2.18 (0.70)	2.50 (0.86)	2.91 (0.93)	2.95 (0.87)	3.06 (1.10)	3.48 (0.75)
10	3.44 (0.94)	3.40 (0.88)	3.37 (0.92)	3.18 (0.66)	3.20 (0.90)	3.24 (1.00)	2.64 (0.82)	2.65 (0.58)	2.83 (0.94)	3.02 (1.01)	3.14 (0.95)	3.54 (1.08)	3.48 (0.76)	3.72 (1.04)	3.99 (0.88)
11	2.37 (0.95)	2.53 (0.84)	2.80 (0.78)	2.41 (0.89)	3.24 (0.64)	3.20 (0.92)	3.25 (0.81)	3.38 (0.87)	3.51 (0.67)	2.44 (0.85)	2.85 (1.08)	3.03 (0.80)	2.74 (0.90)	2.98 (0.78)	2.65 (0.84)
12	3.13 (1.05)	3.46 (0.91)	3.55 (0.87)	3.20 (0.86)	3.07 (0.98)	3.45 (0.77)	2.85 (0.83)	3.04 (0.81)	3.59 (0.58)	3.19 (0.80)	3.39 (0.96)	3.53 (0.95)	2.87 (0.97)	3.05 (0.97)	3.63 (0.90)
13	3.30 (0.81)	3.59 (0.83)	3.52 (0.74)	3.54 (0.65)	3.57 (0.81)	3.70 (0.68)	3.16 (0.73)	3.39 (0.65)	3.60 (0.74)	3.09 (0.92)	3.69 (0.81)	3.66 (0.99)	2.43 (0.79)	3.04 (1.06)	2.92 (0.84)
14	3.32 (1.14)	3.60 (0.71)	3.65 (0.74)	2.74 (1.02)	2.89 (0.67)	2.70 (0.70)	2.58 (0.79)	3.19 (0.77)	3.81 (0.69)	2.58 (0.89)	3.12 (0.97)	3.67 (0.88)	2.49 (0.75)	3.04 (0.83)	3.22 (1.09)
15	3.44 (0.62)	3.09 (0.87)	3.36 (0.77)	3.39 (0.77)	3.33 (0.81)	3.47 (0.79)	2.42 (0.56)	2.80 (0.90)	3.14 (0.65)	2.84 (0.80)	2.94 (0.87)	3.51 (0.85)	3.51 (1.05)	3.17 (0.79)	3.62 (0.87)
16	2.66 (0.80)	2.89 (0.90)	3.07 (1.00)	2.96 (0.69)	2.79 (0.75)	3.04 (0.86)	2.91 (0.79)	3.34 (0.86)	3.73 (0.80)	2.38 (0.79)	3.00 (0.86)	3.48 (1.02)	2.88 (0.90)	3.09 (0.94)	3.29 (0.91)
17	3.15 (1.06)	3.96 (0.60)	3.78 (0.74)	2.74 (0.91)	3.29 (0.82)	3.31 (0.75)	3.07 (0.79)	3.65 (0.58)	3.84 (0.70)	2.95 (0.98)	3.62 (0.85)	3.75 (0.75)	2.29 (0.82)	2.45 (0.73)	3.21 (1.14)
18	3.16 (0.76)	3.18 (0.79)	3.11 (0.70)	3.31 (0.78)	3.33 (0.79)	3.48 (0.68)	2.88 (0.70)	3.19 (0.65)	3.45 (0.75)	3.45 (0.84)	3.29 (0.93)	3.70 (0.91)	2.95 (1.05)	3.34 (0.79)	3.69 (0.86)
19	3.39 (0.75)	3.00 (1.05)	3.38 (0.89)	2.94 (0.78)	2.92 (0.80)	3.02 (0.85)	3.40 (0.79)	3.53 (0.63)	3.83 (0.75)	3.01 (0.96)	3.45 (0.80)	3.86 (1.10)	2.64 (0.89)	2.79 (0.92)	2.31 (0.67)
20	3.08 (0.78)	3.52 (0.76)	3.30 (0.81)	2.77 (0.94)	3.58 (0.72)	3.14 (0.75)	2.82 (0.80)	3.02 (0.68)	3.56 (0.62)	3.03 (0.97)	3.57 (0.96)	3.86 (0.77)	2.53 (0.74)	3.24 (0.96)	3.03 (0.96)
21	2.04 (0.83)	2.36 (0.94)	2.40 (0.84)	2.38 (1.12)	2.39 (0.89)	2.76 (0.92)	2.35 (0.82)	2.70 (0.89)	3.02 (0.65)	2.47 (0.90)	2.58 (0.97)	2.75 (1.11)	3.40 (1.09)	3.38 (0.85)	4.01 (0.84)
22	3.60 (0.82)	3.43 (0.83)	3.46 (0.84)	3.52 (0.66)	3.67 (0.88)	3.42 (0.77)	2.62 (0.57)	3.19 (0.68)	3.08 (0.82)	3.08 (0.88)	3.72 (0.82)	3.45 (0.96)	2.89 (0.75)	2.88 (1.07)	3.37 (0.85)
23	2.51 (0.95)	3.21 (0.66)	2.90 (0.81)	2.77 (0.96)	2.93 (0.84)	3.14 (0.79)	2.66 (0.74)	2.67 (0.82)	3.12 (0.69)	2.65 (0.71)	3.28 (0.97)	3.37 (0.82)	3.83 (0.78)	3.89 (0.76)	4.08 (0.75)
24	2.93 (0.96)	3.45 (0.70)	3.14 (0.74)	3.29 (0.70)	3.21 (0.60)	3.49 (0.63)	3.19 (0.65)	3.62 (0.73)	3.74 (0.70)	3.07 (0.87)	3.36 (0.77)	3.57 (0.83)	2.70 (0.94)	2.81 (0.84)	3.14 (0.89)
25	3.07 (0.94)	2.90 (0.77)	2.92 (0.97)	2.97 (0.75)	3.14 (0.91)	3.05 (0.98)	2.38 (0.78)	2.56 (0.66)	2.83 (0.71)	2.97 (1.01)	3.23 (1.03)	3.61 (0.94)	3.32 (0.75)	3.43 (1.15)	4.04 (0.81)
26	3.15 (0.81)	3.70 (0.85)	3.39 (0.87)	3.30 (0.71)	3.59 (0.64)	3.56 (0.86)	2.50 (0.83)	3.08 (0.75)	3.26 (0.53)	3.30 (0.93)	3.80 (0.86)	3.77 (0.91)	2.79 (0.98)	3.29 (0.83)	3.73 (0.81)
27	2.53 (0.80)	3.11 (0.86)	2.78 (0.87)	3.37 (0.79)	3.56 (0.74)	3.53 (0.74)	2.87 (0.81)	2.88 (0.85)	3.20 (0.85)	2.70 (0.94)	2.95 (1.20)	3.29 (1.14)	3.27 (0.92)	3.19 (0.95)	3.64 (0.83)
28	2.78 (0.92)	2.69 (0.89)	3.05 (0.85)	3.39 (0.81)	3.28 (0.98)	3.24 (1.03)	2.80 (0.72)	3.20 (0.55)	3.36 (0.81)	2.47 (0.81)	3.09 (0.86)	3.26 (0.91)	2.78 (0.75)	2.83 (1.18)	3.13 (0.84)
29	2.80 (0.88)	3.00 (0.85)	3.30 (0.78)	3.29 (0.91)	3.61 (0.67)	3.67 (0.77)	2.86 (0.85)	3.20 (0.61)	3.77 (0.73)	2.52 (0.82)	3.22 (0.86)	3.33 (0.86)	2.35 (0.72)	2.87 (0.94)	3.32 (1.09)
30	2.94 (0.97)	3.21 (1.10)	3.47 (0.85)	2.55 (0.83)	2.38 (0.77)	2.53 (0.91)	3.22 (0.90)	3.41 (1.04)	3.79 (0.76)	2.55 (0.84)	2.83 (0.91)	3.28 (0.94)	2.98 (1.12)	3.24 (0.93)	3.73 (0.81)
31	2.99 (1.03)	3.49 (0.84)	2.98 (1.07)	2.70 (0.92)	2.71 (0.97)	2.51 (0.97)	2.85 (0.87)	3.24 (0.87)	3.27 (0.89)	2.56 (1.04)	2.61 (1.08)	2.85 (1.12)	2.91 (0.95)	3.22 (1.15)	3.67 (0.91)
32	2.65 (0.88)	2.30 (0.91)	3.09 (0.88)	2.85 (0.89)	3.45 (0.65)	3.13 (0.82)	3.05 (0.85)	3.31 (0.73)	3.26 (0.82)	2.44 (0.86)	2.92 (1.12)	3.11 (0.79)	2.45 (0.82)	3.01 (0.87)	3.19 (0.89)
33	2.19 (0.87)	2.26 (0.98)	2.16 (0.87)	2.56 (1.10)	2.41 (0.93)	2.91 (0.85)	2.71 (0.76)	3.26 (0.88)	3.44 (0.76)	2.55 (0.73)	2.57 (0.79)	3.20 (0.99)	2.91 (1.14)	2.89 (0.81)	3.46 (0.83)
34	3.42 (0.86)	2.95 (0.92)	3.25 (0.80)	3.60 (0.84)	3.69 (0.86)	3.45 (0.99)	2.55 (0.71)	2.90 (0.79)	2.89 (0.72)	3.05 (0.93)	3.11 (0.88)	3.18 (0.95)	2.91 (0.94)	3.15 (0.99)	3.05 (0.93)
35	3.28 (0.96)	3.31 (0.75)	3.61 (0.70)	3.01 (0.88)	3.26 (0.72)	3.58 (0.76)	2.97 (0.83)	3.41 (0.76)	3.55 (0.54)	2.68 (0.63)	3.34 (1.01)	3.47 (0.67)	2.92 (0.92)	2.83 (0.76)	3.09 (1.02)
36	3.16 (0.82)	3.00 (1.01)	3.32 (0.69)	2.77 (0.88)	2.93 (0.84)	3.46 (0.66)	2.53 (0.69)	2.51 (0.71)	3.28 (0.74)	2.62 (0.82)	2.84 (0.88)	2.98 (0.81)	3.47 (0.90)	3.60 (1.03)	3.94 (0.67)
37	3.63 (0.63)	3.56 (1.00)	3.64 (0.60)	3.46 (0.64)	3.59 (0.80)	3.52 (0.73)	3.10 (0.69)	3.60 (0.71)	3.74 (0.66)	3.05 (1.00)	3.58 (0.79)	3.71 (0.94)	2.65 (0.86)	3.08 (1.17)	2.98 (1.05)
38	3.09 (0.90)	3.18 (0.80)	3.78 (0.74)	2.96 (0.90)	3.49 (0.67)	3.42 (0.86)	2.75 (0.82)	3.36 (0.76)	3.53 (0.71)	2.45 (0.86)	3.16 (0.90)	3.69 (0.79)	2.79 (0.75)	2.88 (0.90)	3.49 (1.00)
39	2.92 (0.92)	3.03 (0.86)	3.14 (0.89)	3.10 (0.81)	3.20 (0.78)	3.35 (0.76)	2.82 (0.77)	3.25 (0.79)	3.50 (0.68)	3.18 (0.77)	3.16 (0.83)	3.29 (0.78)	3.37 (1.00)	3.39 (0.86)	3.59 (0.80)
40	3.04 (0.78)	2.74 (0.81)	3.04 (0.88)	3.41 (0.63)	3.35 (0.85)	3.06 (0.78)	2.30 (0.80)	2.27 (0.72)	2.58 (0.76)	2.30 (0.80)	2.99 (0.83)	3.03 (1.11)	3.51 (0.66)	3.81 (1.02)	4.04 (0.83)
all	2.96 (1.16)	3.16 (1.14)	3.26 (1.08)	3.01 (1.10)	3.15 (1.08)	3.22 (1.08)	2.83 (0.99)	3.15 (0.99)	3.41 (0.96)	2.79 (1.13)	3.18 (1.17)	3.42 (1.16)	2.91 (1.14)	3.10 (1.16)	3.38 (1.19)

### Discussion

Study 2 suggests that the 40 faces were successfully manipulated regarding the Big Five dimensions openness, conscientiousness, extraversion, agreeableness, and neuroticism. As in Study 1, manipulation success generalizes across the different faces. Nevertheless, we provide descriptive data for the 40 individual faces separately, so that the most promising identities for a given purpose can be systematically selected.

## General discussion

This contribution presents a new face database consisting of 40 different facial identities systematically modeled regarding the Big Two and the Big Five personality dimensions. Results of two studies suggest that the seven personality dimensions were successfully manipulated in real face photographs. In particular, participants reliably detected changes on each of the manipulated dimensions, evaluating, for instance, a face with an enhanced compared to a reduced value on extraversion as more extraverted. The Basel Face Database (BFD) thus allows researchers to independently manipulate each of the Big Two or Big Five personality dimensions with portraits derived from real photographs. This is important for all types of research projects in which the perception of one isolated aspect of personality and its consequences on judgments and actions is addressed. Researchers can now present the same face, manipulated only regarding the cues associated with the one personality dimension in question, to participants in different conditions of the study. Changes in the dependent variable can then be attributed to systematic differences in the independent variable, resulting in high *internal validity*.

Comparing data across the two studies, manipulations on the Big Two dimensions yielded stronger effects on personality inferences than manipulations on the Big Five dimensions. This finding is in line with previous work showing that trustworthiness and dominance, the two dimensions that highly overlap with communion and agency, are fundamental dimensions in person evaluations from faces [21]. Despite this relative difference, however, manipulations of all Big Five dimensions were successful as well, providing researchers with a valuable resource to investigate research questions pertaining to the Big Five personality model, too. We have opted to present data on the stimulus level so that researchers may choose according to the respective needs of their study. At the same time, we encourage researchers to rely on large sets of BFD faces, so as to harvest the benefits of experimental designs with crossed random effects for targets and participants (i.e., within-target and within-participant manipulations). Such data are suitable for linear mixed models analyses [39], and enhance replicability of the findings and generalizability across targets, ultimately resulting in high *external validity*.

One distinctive feature of the BFD is that it is based on photographs of real faces, thereby enhancing *ecological validity*. This feature is important in contexts in which photographs of faces are preferable compared to avatars (e.g., stereotyping research, [44]). However, there are also contexts in which avatars are highly desirable and suitable. Researchers may therefore choose the stimuli based on their research question.

We have suggested that the BFD can be used to investigate the *impact of facial cues* associated with a specific personality dimension on different outcome variables. However, because a large set of faces is provided, researchers may also more generally investigate the *impact of personality* on outcome variables. That the BFD faces do not obviously reveal that they are computer-generated may be a particularly conducive feature in this respect, because they are unlikely to interfere with the judgment or decision process and serve the goal to introduce personality in a very inconspicuous way. What might be perceived as a mere illustration to render the study material more realistic in fact can serve as the operationalization of the independent variable, personality.

### Limitations and future research

The 40 original face photographs are rather homogeneous regarding the age and cultural background of the depicted persons. One goal for future research is to enrich this database by adding photographs of other age groups and other cultural backgrounds. It has been shown before that our models can be successfully applied to faces from different cultural backgrounds [45].

### Conclusion

The Basel Face Database (BFD) consists of 40 different facial identities systematically modeled on the Big Two and the Big Five personality dimensions. Because the BFD models dimensions independently and uses subtle but systematic variations of real face photographs, it may be fruitfully relied on by researchers in various fields, including (but not limited to) social, law, consumer, economic, personality, moral, or clinical psychology. The BFD is freely available to researchers and can be requested here: bfd.unibas.ch.

## Supporting information

S1 Supporting Information Originality/manipulatedness item (Study 1 and Study 2). (DOCX)
