# Supplementary material for: The Basel Face Database: A validated set of photographs reflecting systematic differences in Big Two and Big Five personality dimensions
Source: PLoS One. 2018 Mar 28;13(3):e0193190. doi: 10.1371/journal.pone.0193190 (PMC5873939; doi:10.1371/journal.pone.0193190)
Supplement: S1 Supporting Information — (DOCX) [file pone.0193190.s001.docx]

**S1 Supporting Information**

**Originality/manipulatedness item (Study 1 and Study 2)**

After debriefing participants that some photographs they had seen were real photographs, while others had been systematically altered to depict another personality, participants judged each portrait on a 4-point scale: 1 = “certainly an original photograph”, 2 = “rather an original photograph than a manipulated version”, 3 = “rather a manipulated version than an original photograph”, or 4 = “certainly a manipulated version”. This data is part of different project that investigates to what degree the perceived averageness/uniqueness of personality impacts perceptions of manipulatedness of photographs. However, the data may also be of interest here, since it provides information about whether the photographs are perceived as original or manipulated, *given* that participants are explicitly informed that some photographs were manipulated. Note that this constraint is critical, since perceptions of manipulatedness may result from this instruction, and not from cues inherent to the photographs. Nevertheless, in study contexts where participants may be suspicious (e.g., studies focusing on lie detection), knowing about this data on the level of individual faces (see Tables 1 and 2) may prove fruitful. Interestingly, despite the explicit warning that some photographs were manipulated, all photographs are perceived as rather original than manipulated.

We analyzed the results for this originality/manipulatedness item in both studies by building three different models each: A random model allowing participants and faces to show individual intercepts and slopes for face value and additionally allowing faces to show individual slopes for different personality dimensions, a model with the additional fixed factor face value and a model with the additional interaction effect between face value and personality dimension. Model fit and model comparisons for both studies are depicted in Table 3.

Model comparisons in both studies reveal that manipulating the value of faces on different personality dimensions – independent of the kind of personality dimension – has an impact on perceived originality/manipulatedness, given that participants were made aware that some photographs have been digitally altered. The manipulated versions (reduced value: *M_Study1_* = 2.48, *SE _Study1_* = .0.05 and *M_Study2_* = 2.27, *SE_Study2_* = 0.04, respectively, and enhanced value: *M_Study1_* = 2.26, *SE_Study1_* = 0.06 and *M_Study2_* = 2.24, *SE_Study2_* = 0.04) were perceived as less original than the original versions (*M_Study1_* = 2.03, *SE_Study1_* = 0.05 and *M_Study2_* = 2.16, *SE_Study2_* = 0.04).

Although mean values of originality/manipulatedness differ between the versions with reduced, original, and enhanced value, this difference is rather small with mean ratings for all versions, original and manipulated ones, ranging from 1.99 (with 2 equaling “rather an original photograph than a manipulated version”) to 2.53 (i.e., around the scale midpoint) on a scale ranging from 1 to 5. Of interest, the original faces were also not classified as “certainly an original photograph” (i.e., the scale endpoint), suggesting that there may not be something that rendered the manipulated faces particularly manipulated, but that participants overall had a high threshold for indicating “original.” Moreover, given the instruction that some photographs have been manipulated, it is unsurprising that participants judged some photographs to be manipulated. Without explicitly telling participants that some photographs have been digitally altered, we expect doubts about originality to be much lower. We note that this hunch awaits empirical substantiation, and that researchers concerned about originality/manipulatedness might discard the few manipulated faces which were on average rated as rather being a manipulated than an original photograph. To allow this selection, we provide the originality/manipulatedness data separately for all 40 faces in Tables 1 and 2.

Table 1

Means and standard deviations of the originality/manipulatedness judgments for the 40 facial identities with reduced, original, and enhanced value on both Big Two personality dimensions.

| ID | | Agency | | | | Communion | | |
| --- | --- | --- | --- | --- | --- | --- | --- | --- |
|  | -  M(SD) | | -/+  M(SD) | +  M(SD) | -  M(SD) | | -/+  M(SD) | +  M(SD) |
| 1 | 2.72 (1.02) | | 2.01 (0.97) | 2.21 (0.96) | 2.64 (1.09) | | 2.16 (1.07) | 2.50 (1.19) |
| 2 | 2.51 (0.91) | | 1.85 (0.86) | 2.10 (1.07) | 2.40 (0.95) | | 1.64 (0.81) | 1.68 (0.74) |
| 3 | 2.12 (1.08) | | 1.99 (0.80) | 1.85 (0.73) | 2.42 (1.01) | | 2.05 (0.91) | 2.13 (0.97) |
| 4 | 2.85 (0.98) | | 2.07 (1.00) | 1.90 (0.88) | 2.30 (1.05) | | 1.91 (0.85) | 2.43 (1.06) |
| 5 | 2.26 (0.85) | | 1.92 (0.79) | 2.32 (0.96) | 2.29 (1.04) | | 2.13 (1.11) | 2.35 (0.92) |
| 6 | 2.32 (0.96) | | 1.74 (0.73) | 1.75 (0.68) | 2.09 (0.68) | | 1.67 (0.82) | 1.50 (0.75) |
| 7 | 2.32 (0.96) | | 2.12 (0.93) | 2.74 (0.94) | 2.76 (1.27) | | 1.94 (0.73) | 2.09 (1.00) |
| 8 | 2.49 (0.78) | | 1.99 (0.91) | 2.32 (0.90) | 2.12 (0.86) | | 1.70 (0.76) | 1.94 (0.83) |
| 9 | 2.04 (0.99) | | 1.96 (0.88) | 1.79 (0.76) | 2.42 (0.93) | | 1.67 (0.72) | 1.96 (0.94) |
| 10 | 2.99 (1.05) | | 2.65 (1.02) | 2.68 (0.92) | 3.07 (1.11) | | 2.31 (1.07) | 2.67 (1.01) |
| 11 | 3.04 (0.86) | | 2.05 (0.91) | 2.49 (1.09) | 2.74 (1.00) | | 2.10 (1.13) | 2.39 (0.97) |
| 12 | 2.49 (1.06) | | 1.87 (0.89) | 2.12 (0.78) | 2.42 (1.01) | | 1.81 (0.98) | 2.24 (1.14) |
| 13 | 1.95 (0.89) | | 1.71 (0.78) | 2.21 (0.90) | 2.79 (1.12) | | 1.76 (0.72) | 2.09 (0.93) |
| 14 | 2.51 (0.78) | | 1.95 (0.85) | 2.21 (0.93) | 2.53 (0.79) | | 1.64 (0.91) | 2.24 (1.05) |
| 15 | 2.35 (0.93) | | 2.32 (0.96) | 2.22 (0.86) | 2.50 (1.06) | | 2.02 (0.93) | 2.24 (1.01) |
| 16 | 2.22 (0.78) | | 1.96 (0.91) | 1.96 (0.91) | 2.22 (1.11) | | 1.79 (0.80) | 2.36 (0.86) |
| 17 | 2.35 (0.90) | | 2.25 (1.02) | 2.18 (1.01) | 3.22 (0.98) | | 2.04 (0.89) | 2.05 (0.86) |
| 18 | 2.07 (0.97) | | 1.90 (0.73) | 1.92 (0.74) | 1.91 (0.90) | | 1.88 (0.88) | 1.87 (0.85) |
| 19 | 2.55 (0.73) | | 2.32 (1.10) | 2.99 (0.87) | 3.13 (1.06) | | 2.35 (0.74) | 2.12 (1.12) |
| 20 | 2.68 (0.79) | | 2.05 (0.83) | 2.04 (1.02) | 2.50 (1.03) | | 1.79 (1.01) | 2.13 (0.85) |
| 21 | 2.18 (0.75) | | 2.15 (0.85) | 1.89 (0.71) | 1.83 (0.88) | | 1.81 (0.94) | 2.02 (0.94) |
| 22 | 2.22 (0.86) | | 2.35 (1.08) | 2.96 (0.97) | 2.64 (1.12) | | 2.42 (0.98) | 2.64 (1.02) |
| 23 | 2.40 (0.88) | | 1.99 (0.95) | 1.96 (0.91) | 2.40 (0.95) | | 2.04 (0.93) | 1.72 (0.73) |
| 24 | 2.37 (0.97) | | 1.82 (0.81) | 1.89 (0.81) | 2.65 (0.84) | | 1.84 (0.85) | 2.44 (1.10) |
| 25 | 2.72 (0.79) | | 1.79 (0.89) | 2.01 (0.91) | 2.30 (1.05) | | 1.87 (0.82) | 2.53 (0.87) |
| 26 | 2.57 (0.81) | | 1.95 (0.81) | 2.21 (0.87) | 2.81 (0.86) | | 1.62 (0.85) | 2.20 (0.84) |
| 27 | 2.01 (0.97) | | 2.07 (0.88) | 2.92 (0.79) | 2.53 (0.90) | | 2.09 (0.97) | 2.82 (1.00) |
| 28 | 1.89 (0.61) | | 1.57 (0.73) | 2.10 (0.86) | 2.56 (1.07) | | 1.79 (0.94) | 2.12 (0.86) |
| 29 | 2.46 (1.00) | | 2.09 (0.71) | 2.10 (1.07) | 2.26 (0.95) | | 2.04 (1.02) | 2.50 (0.90) |
| 30 | 2.76 (1.05) | | 2.79 (0.95) | 3.29 (0.71) | 2.68 (0.96) | | 2.81 (0.98) | 3.16 (0.98) |
| 31 | 2.62 (0.97) | | 2.10 (1.01) | 2.54 (1.06) | 2.04 (1.21) | | 2.57 (1.05) | 2.88 (1.10) |
| 32 | 2.62 (0.90) | | 2.05 (0.87) | 2.04 (0.93) | 2.33 (0.97) | | 1.99 (0.93) | 2.28 (0.77) |
| 33 | 2.32 (1.07) | | 1.96 (0.88) | 1.55 (0.73) | 1.98 (0.94) | | 2.05 (0.91) | 2.13 (0.94) |
| 34 | 2.82 (1.15) | | 2.26 (1.01) | 2.29 (0.95) | 2.93 (1.13) | | 2.02 (0.92) | 2.36 (1.05) |
| 35 | 2.57 (1.03) | | 2.25 (0.91) | 2.21 (1.02) | 2.40 (0.95) | | 1.84 (0.89) | 2.50 (0.85) |
| 36 | 2.29 (0.92) | | 2.40 (0.84) | 2.45 (0.82) | 2.72 (1.14) | | 2.12 (0.82) | 2.13 (0.94) |
| 37 | 2.59 (1.14) | | 2.21 (1.02) | 2.24 (0.84) | 2.70 (1.08) | | 1.94 (0.83) | 2.43 (1.06) |
| 38 | 2.51 (1.00) | | 2.09 (0.76) | 2.29 (1.01) | 2.67 (0.90) | | 1.99 (0.86) | 1.91 (0.85) |
| 39 | 2.96 (0.91) | | 2.54 (1.00) | 3.12 (0.78) | 3.05 (0.94) | | 2.57 (0.99) | 2.64 (1.09) |
| 40 | 2.12 (0.73) | | 1.99 (0.83) | 2.10 (0.92) | 2.67 (1.11) | | 1.94 (0.83) | 2.09 (0.80) |
| all | 2.44 (1.17) | | 2.08 (1.12) | 2.25 (1.18) | 2.53 (1.28) | | 1.99 (1.14) | 2.26 (1.22) |

Table 2

Means and standard deviations of the originality/manipulatedness judgments for the 40 facial identities with reduced, original, and enhanced value on all Big Five personality dimensions.

| ID | Openness | | | Conscientiousness | | | Extraversion | | | Agreeableness | | | Neuroticism | | |
| --- | --- | --- | --- | --- | --- | --- | --- | --- | --- | --- | --- | --- | --- | --- | --- |
|  | -  M(SD) | -/+  M(SD) | +  M(SD) | -  M(SD) | -/+  M(SD) | +  M(SD) | -  M(SD) | -/+  M(SD) | +  M(SD) | -  M(SD) | -/+  M(SD) | +  M(SD) | -  M(SD) | -/+  M(SD) | +  M(SD) |
| 1 | 2.24 (1.13) | 2.36 (1.20) | 2.58 (1.01) | 2.40 (0.86) | 2.16 (0.96) | 2.47 (0.93) | 2.36 (1.04) | 2.65 (0.91) | 1.96 (1.06) | 2.57 (0.98) | 2.44 (0.98) | 2.29 (0.91) | 2.27 (0.98) | 2.07 (1.15) | 2.78 (0.99) |
| 2 | 2.10 (1.01) | 1.90 (0.90) | 1.72 (0.77) | 2.20 (0.87) | 2.06 (0.83) | 1.91 (0.82) | 1.99 (0.96) | 2.25 (0.98) | 1.85 (0.85) | 2.29 (0.79) | 2.01 (0.79) | 1.92 (0.72) | 1.94 (1.05) | 2.07 (0.93) | 2.07 (0.92) |
| 3 | 1.75 (0.75) | 1.90 (0.96) | 2.35 (0.90) | 2.05 (0.94) | 1.94 (0.75) | 2.17 (0.88) | 2.16 (0.93) | 1.79 (0.73) | 2.19 (0.97) | 2.15 (0.84) | 2.14 (0.79) | 2.13 (0.92) | 1.81 (0.97) | 1.98 (0.97) | 2.01 (0.90) |
| 4 | 2.48 (0.95) | 2.07 (0.98) | 2.23 (1.10) | 1.90 (0.82) | 2.16 (0.96) | 2.47 (0.90) | 2.11 (0.97) | 2.39 (0.92) | 2.02 (0.97) | 2.18 (0.97) | 2.12 (0.95) | 2.14 (0.92) | 2.04 (0.91) | 1.99 (1.07) | 2.24 (0.96) |
| 5 | 2.48 (0.85) | 2.14 (0.92) | 2.54 (1.08) | 2.31 (0.90) | 2.17 (1.09) | 2.33 (0.95) | 2.40 (1.11) | 2.25 (0.95) | 2.31 (1.00) | 2.44 (1.00) | 2.23 (0.98) | 2.41 (0.85) | 2.74 (0.93) | 2.07 (1.00) | 2.38 (1.17) |
| 6 | 2.11 (0.88) | 1.98 (0.95) | 1.90 (0.82) | 2.10 (0.97) | 1.84 (0.69) | 1.83 (0.70) | 2.34 (0.88) | 1.90 (0.97) | 2.02 (0.86) | 2.24 (0.94) | 2.04 (0.80) | 1.74 (0.75) | 2.07 (1.10) | 1.98 (0.81) | 2.10 (0.90) |
| 7 | 2.83 (0.97) | 2.04 (0.84) | 2.10 (1.11) | 2.21 (1.02) | 2.24 (0.99) | 2.15 (0.91) | 2.28 (1.05) | 2.25 (0.86) | 2.20 (0.95) | 2.47 (0.93) | 2.21 (0.86) | 2.04 (0.92) | 2.56 (0.92) | 2.29 (1.27) | 2.04 (0.93) |
| 8 | 1.95 (0.75) | 1.97 (0.78) | 1.97 (0.88) | 2.13 (0.80) | 1.98 (0.83) | 2.10 (0.84) | 1.85 (0.93) | 1.99 (0.91) | 2.19 (0.97) | 2.24 (0.90) | 1.82 (0.73) | 2.21 (0.86) | 1.94 (1.02) | 2.07 (0.96) | 2.03 (1.00) |
| 9 | 1.93 (0.98) | 1.75 (0.84) | 2.00 (0.76) | 1.99 (0.85) | 2.07 (0.87) | 2.14 (0.82) | 2.11 (0.90) | 2.11 (0.88) | 2.08 (0.95) | 2.18 (0.74) | 2.19 (0.82) | 2.08 (0.84) | 1.90 (0.93) | 1.71 (0.82) | 1.81 (0.93) |
| 10 | 2.76 (1.06) | 2.50 (1.24) | 2.15 (1.13) | 2.79 (1.11) | 2.10 (0.97) | 2.70 (0.88) | 2.79 (1.06) | 2.82 (0.93) | 2.73 (1.17) | 2.67 (1.14) | 2.41 (0.91) | 2.54 (0.89) | 2.36 (0.85) | 2.38 (1.28) | 2.74 (0.89) |
| 11 | 2.35 (1.03) | 2.42 (0.95) | 2.11 (1.07) | 2.49 (0.98) | 2.29 (0.83) | 2.33 (0.89) | 2.29 (0.97) | 2.45 (0.92) | 2.48 (1.02) | 2.74 (0.74) | 2.28 (1.00) | 2.29 (1.04) | 2.08 (0.78) | 2.20 (0.97) | 2.33 (1.16) |
| 12 | 2.11 (0.92) | 2.10 (1.13) | 2.42 (1.12) | 2.13 (1.09) | 2.10 (0.85) | 2.33 (0.93) | 2.25 (1.04) | 2.49 (1.14) | 2.36 (1.09) | 1.81 (0.69) | 2.09 (0.83) | 2.47 (1.01) | 2.33 (1.20) | 2.28 (1.07) | 2.33 (1.06) |
| 13 | 2.52 (1.03) | 1.68 (0.87) | 1.73 (0.94) | 1.98 (0.72) | 1.83 (0.86) | 2.05 (1.06) | 2.02 (0.93) | 2.08 (0.92) | 1.61 (0.93) | 2.11 (0.94) | 2.01 (0.89) | 1.79 (0.85) | 2.07 (0.85) | 2.03 (1.05) | 2.21 (0.87) |
| 14 | 2.18 (1.09) | 1.90 (0.94) | 1.79 (0.79) | 2.28 (0.89) | 2.14 (0.91) | 2.16 (0.90) | 1.96 (0.91) | 2.28 (0.96) | 1.99 (0.88) | 2.24 (0.84) | 2.04 (0.90) | 2.15 (0.90) | 2.11 (0.95) | 2.33 (0.89) | 2.07 (0.97) |
| 15 | 2.25 (0.89) | 2.13 (1.00) | 2.00 (0.97) | 2.08 (1.02) | 2.23 (0.94) | 2.17 (0.93) | 2.39 (0.92) | 2.14 (0.96) | 2.08 (0.92) | 2.58 (0.98) | 2.04 (0.80) | 2.28 (0.97) | 2.16 (1.00) | 1.78 (0.79) | 2.52 (0.81) |
| 16 | 1.97 (0.73) | 1.86 (0.81) | 2.08 (1.03) | 2.02 (0.94) | 2.05 (0.91) | 2.18 (0.89) | 2.19 (0.94) | 2.02 (0.86) | 1.76 (0.74) | 2.18 (0.94) | 1.92 (0.76) | 2.24 (0.77) | 2.01 (0.82) | 1.86 (1.09) | 2.18 (0.86) |
| 17 | 2.35 (1.08) | 2.24 (0.92) | 1.79 (0.74) | 2.57 (0.95) | 2.17 (0.97) | 2.41 (0.91) | 2.23 (0.89) | 2.16 (0.99) | 2.05 (0.88) | 2.69 (0.94) | 2.11 (0.88) | 2.01 (0.75) | 2.44 (0.82) | 2.14 (0.80) | 2.42 (0.95) |
| 18 | 2.11 (0.96) | 2.00 (1.14) | 2.28 (0.75) | 2.44 (0.88) | 1.92 (0.83) | 1.79 (0.71) | 1.85 (0.65) | 1.82 (0.80) | 1.94 (0.91) | 2.12 (0.99) | 2.04 (0.80) | 1.79 (0.63) | 2.16 (1.00) | 1.64 (0.88) | 1.98 (0.88) |
| 19 | 2.48 (0.91) | 2.43 (1.00) | 2.30 (0.97) | 2.64 (1.07) | 2.49 (0.88) | 2.44 (0.80) | 2.54 (0.95) | 2.36 (0.95) | 2.52 (0.93) | 2.60 (0.90) | 2.44 (0.88) | 2.34 (1.13) | 2.49 (0.93) | 2.60 (0.94) | 2.34 (1.11) |
| 20 | 2.28 (0.88) | 2.35 (0.86) | 2.29 (0.90) | 2.20 (0.80) | 2.10 (0.80) | 2.27 (0.88) | 2.29 (1.06) | 2.25 (1.04) | 2.48 (0.99) | 2.54 (0.89) | 2.21 (0.95) | 2.18 (0.85) | 1.98 (0.85) | 2.07 (0.93) | 2.07 (1.06) |
| 21 | 1.68 (0.82) | 1.90 (0.99) | 2.21 (0.91) | 2.02 (0.90) | 1.97 (0.84) | 2.14 (0.77) | 2.11 (0.87) | 1.76 (0.82) | 2.14 (0.95) | 2.32 (1.05) | 2.34 (0.80) | 2.08 (0.90) | 2.03 (1.05) | 2.08 (0.98) | 2.33 (0.92) |
| 22 | 2.55 (0.95) | 2.43 (1.11) | 2.55 (1.07) | 2.64 (0.95) | 2.46 (0.97) | 2.28 (0.98) | 2.56 (0.98) | 2.56 (1.01) | 2.40 (1.08) | 2.47 (1.05) | 2.58 (0.86) | 2.49 (1.05) | 2.69 (1.04) | 2.25 (1.19) | 2.31 (0.93) |
| 23 | 2.08 (1.00) | 1.97 (0.91) | 1.82 (0.95) | 2.07 (0.81) | 1.83 (0.75) | 1.99 (0.85) | 1.96 (0.87) | 2.25 (0.95) | 1.99 (0.88) | 2.04 (0.65) | 1.84 (0.75) | 1.98 (0.91) | 2.14 (0.89) | 2.14 (0.88) | 2.20 (1.14) |
| 24 | 2.32 (0.95) | 1.88 (0.83) | 2.35 (0.86) | 2.24 (0.84) | 1.97 (0.84) | 1.87 (0.73) | 2.19 (0.94) | 1.99 (0.92) | 1.96 (0.89) | 1.86 (0.79) | 1.99 (0.89) | 1.99 (0.84) | 1.94 (0.86) | 2.04 (0.85) | 2.14 (0.71) |
| 25 | 2.42 (1.06) | 1.72 (0.77) | 2.53 (0.99) | 2.02 (0.94) | 2.35 (0.83) | 2.31 (0.99) | 2.22 (0.91) | 2.25 (1.04) | 2.05 (0.89) | 2.06 (0.94) | 2.35 (0.94) | 2.19 (0.75) | 1.98 (0.80) | 2.16 (0.85) | 2.58 (0.97) |
| 26 | 2.30 (0.94) | 2.17 (0.89) | 2.11 (1.00) | 2.34 (0.88) | 1.90 (0.82) | 2.16 (0.93) | 2.20 (0.92) | 1.85 (0.85) | 2.11 (0.90) | 2.14 (0.79) | 1.84 (0.78) | 2.06 (0.79) | 1.81 (0.94) | 2.20 (0.82) | 2.38 (1.04) |
| 27 | 2.82 (0.95) | 2.13 (0.97) | 2.55 (0.91) | 2.35 (0.83) | 2.10 (0.94) | 2.56 (0.95) | 2.39 (1.01) | 2.05 (0.92) | 2.39 (0.95) | 2.35 (0.84) | 2.34 (0.87) | 2.30 (0.95) | 2.42 (1.04) | 2.24 (0.84) | 2.59 (0.66) |
| 28 | 1.86 (0.75) | 1.89 (0.74) | 1.90 (0.78) | 2.44 (0.86) | 1.80 (0.72) | 1.92 (0.83) | 2.16 (0.89) | 1.82 (0.83) | 2.02 (1.00) | 1.96 (0.82) | 2.04 (0.81) | 1.84 (0.77) | 2.27 (0.91) | 2.03 (1.00) | 1.98 (0.81) |
| 29 | 2.20 (0.97) | 2.14 (0.88) | 2.39 (1.03) | 2.34 (0.97) | 2.21 (0.98) | 2.21 (0.92) | 2.29 (1.01) | 2.05 (0.81) | 2.05 (1.03) | 2.24 (0.77) | 2.06 (0.88) | 2.38 (1.00) | 2.14 (0.89) | 1.98 (0.71) | 2.16 (1.09) |
| 30 | 3.00 (1.02) | 2.68 (1.00) | 2.69 (1.01) | 2.85 (0.95) | 2.94 (0.82) | 2.79 (1.04) | 2.68 (0.94) | 2.67 (1.13) | 2.79 (1.03) | 2.75 (1.08) | 2.64 (1.00) | 2.82 (0.97) | 2.64 (0.88) | 2.84 (0.96) | 2.85 (0.91) |
| 31 | 2.62 (1.18) | 2.32 (1.10) | 2.40 (1.09) | 2.02 (0.85) | 2.21 (1.01) | 2.63 (1.01) | 2.62 (0.85) | 2.28 (1.02) | 2.43 (0.99) | 2.21 (1.03) | 2.55 (1.13) | 2.64 (0.76) | 2.43 (1.03) | 2.20 (1.06) | 2.68 (0.95) |
| 32 | 2.10 (0.93) | 2.73 (0.89) | 2.25 (0.85) | 2.15 (0.91) | 2.21 (0.98) | 2.16 (0.90) | 2.17 (0.84) | 2.19 (1.06) | 2.22 (0.88) | 2.44 (0.89) | 2.13 (0.92) | 2.32 (0.86) | 2.51 (1.04) | 2.36 (0.93) | 2.42 (1.28) |
| 33 | 1.93 (1.02) | 1.88 (0.89) | 1.97 (1.02) | 1.83 (0.75) | 2.05 (0.89) | 2.02 (0.85) | 2.28 (0.83) | 1.70 (0.68) | 2.11 (0.84) | 1.92 (0.84) | 1.99 (0.61) | 2.21 (0.92) | 1.86 (0.85) | 1.58 (0.85) | 2.04 (0.98) |
| 34 | 2.76 (0.99) | 2.29 (1.02) | 2.53 (1.04) | 2.17 (0.97) | 2.38 (1.16) | 2.49 (0.96) | 2.31 (0.94) | 2.54 (0.95) | 2.46 (1.08) | 2.47 (1.03) | 2.29 (1.04) | 2.54 (1.06) | 2.27 (0.98) | 2.29 (0.99) | 2.21 (0.98) |
| 35 | 2.13 (1.05) | 2.35 (1.01) | 2.36 (1.17) | 2.15 (0.94) | 2.44 (0.95) | 2.27 (0.97) | 2.32 (0.98) | 2.22 (0.91) | 2.14 (0.88) | 2.44 (0.82) | 2.08 (0.84) | 2.38 (0.94) | 2.01 (0.87) | 2.20 (0.97) | 2.38 (1.08) |
| 36 | 2.39 (1.07) | 2.10 (0.90) | 2.31 (0.93) | 2.19 (1.03) | 2.15 (1.10) | 2.29 (0.83) | 2.62 (1.01) | 1.99 (0.89) | 2.31 (0.94) | 2.38 (0.88) | 1.84 (0.53) | 1.86 (0.90) | 2.16 (1.13) | 2.14 (0.97) | 2.43 (0.81) |
| 37 | 2.21 (0.82) | 2.04 (0.97) | 2.25 (1.01) | 2.60 (0.86) | 2.19 (0.88) | 1.99 (0.83) | 2.16 (0.99) | 2.08 (1.02) | 2.35 (0.95) | 2.40 (1.00) | 1.98 (0.88) | 2.34 (0.98) | 2.30 (1.02) | 2.55 (0.99) | 2.54 (1.04) |
| 38 | 2.55 (1.18) | 2.93 (0.93) | 2.07 (0.90) | 2.15 (0.94) | 2.33 (0.93) | 2.35 (0.90) | 2.32 (1.07) | 2.08 (0.89) | 2.36 (0.88) | 2.39 (0.94) | 2.33 (0.90) | 2.24 (0.90) | 2.48 (0.94) | 2.20 (1.00) | 2.16 (1.21) |
| 39 | 3.07 (0.86) | 2.43 (0.93) | 2.86 (0.96) | 2.66 (1.08) | 2.52 (1.01) | 2.75 (0.92) | 2.65 (0.94) | 2.88 (0.81) | 2.79 (1.06) | 2.72 (1.03) | 2.74 (0.98) | 2.62 (1.08) | 2.73 (1.09) | 2.71 (1.01) | 2.98 (0.80) |
| 40 | 2.17 (0.93) | 2.22 (0.96) | 2.43 (0.96) | 2.33 (0.79) | 2.02 (0.93) | 2.47 (0.83) | 2.31 (1.03) | 1.85 (0.85) | 2.26 (0.97) | 2.08 (0.95) | 2.06 (0.79) | 1.99 (0.76) | 2.17 (0.77) | 2.12 (1.03) | 2.18 (0.98) |
| all | 2.30 (1.22) | 2.14 (1.20) | 2.23 (1.21) | 2.26 (1.14) | 2.15 (1.13) | 2.26 (1.11) | 2.27 (1.17) | 2.19 (1.17) | 2.20 (1.19) | 2.31 (1.15) | 2.16 (1.09) | 2.20 (1.12) | 2.21 (1.18) | 2.14 (1.19) | 2.31 (1.20) |

Table 3

AICs for the different linear mixed models with the same random effects as specified in the analyses in the main manuscript and the dependent variable originality/manipulatedness of the photograph and c2 and p-values for model comparisons for both studies.

| **Study 1** | AIC | c^2^ | df | p |
| --- | --- | --- | --- | --- |
| 1) Random model | 20104 |  |  |  |
| 2) Fixed effect face value model | 20040 | 67.46 | 2 | <.001 |
| 3) Interaction model (face value x personality dimension) | 20039 | 7.01 | 3 | .072 |
| **Study 2** |  |  |  |  |
| 1) Random model | 49345 |  |  |  |
| 2) Fixed effect face value model | 49313 | 35.24 | 2 | <.001 |
| 3) Interaction model (face value x personality dimension) | 49316 | 21.23 | 12 | .047 |
